# Correction: Iron tris-mesityl: a homoleptic iron(ii) ferrate species for directed C–H activation

**DOI:** 10.1039/d6sc90055k

**Published:** 2026-02-27

**Authors:** Aleksa Radović, Maria C. Healy, Arnadeep Datta, Deborshee Das, Likun Cai, Steven Diaz, Achyut Ranjan Gogoi, Nikki J. Wolford, Stephanie H. Carpenter, William W. Brenessel, David McCamant, Osvaldo Gutierrez, Michael L. Neidig

**Affiliations:** a Department of Chemistry, University of Rochester 120 Trustee Rd Rochester NY14627 USA; b Department of Chemistry, Inorganic Chemistry Laboratory, University of Oxford South Parks Road Oxford OX1 3QR UK; c Department of Chemistry and Biochemistry, University of California, Los Angeles 607 Charles E. Young Drive East Los Angeles CA 90095 USA o.gutierrez@ucla.edu

## Abstract

Correction for ‘Iron tris-mesityl: a homoleptic iron(ii) ferrate species for directed C–H activation’ by Aleksa Radović *et al.*, *Chem. Sci.*, 2026, https://doi.org/10.1039/d5sc08832a.

The authors regret that ref. 31 in their published article refers to the German version as opposed to the international version of *Angewandte Chemie*.

The correct, international version of ref. 31 is given below as ref. 1.

The Royal Society of Chemistry apologises for these errors and any consequent inconvenience to authors and readers.
